# Radiological findings in pelvic solitary fibrous tumour

**DOI:** 10.1259/bjrcr.20150373

**Published:** 2016-11-02

**Authors:** Paul Johannet, Aya Kamaya, Gabriela Gayer

**Affiliations:** Department of Radiology, Stanford University, Stanford, CA, USA

## Abstract

Solitary fibrous tumour (SFT) is an uncommon, usually benign mesenchymal neoplasm. SFT was first described in the pleura, but has subsequently been reported to occur in numerous anatomic locations including the abdomen and pelvis. Abdominopelvic SFTs are typically an indolent process, in spite of reaching a large size by the time of diagnosis. The preferred treatment is complete resection followed by extended follow-up surveillance. The risk of local recurrence and metastasis correlates with tumour size and the histological status of surgical margins. We present the imaging findings of a large pelvic SFT in a 61-year-old female, including ultrasound, CT and MRI.

## Clinical presentation

A 61-year-old female presented with 4 months of intermittent, non-radiating lower back pain that she attributed to her long-standing scoliosis. She denied concomitant abdominal pain, constipation, difficulty in urinating, blood in her stool or urine and changes in her appetite or weight. On physical examination, the patient was found to have a pelvic mass. The remainder of the history and physical examination was non-contributory.

## Investigations/imaging findings

**Figure 1.**
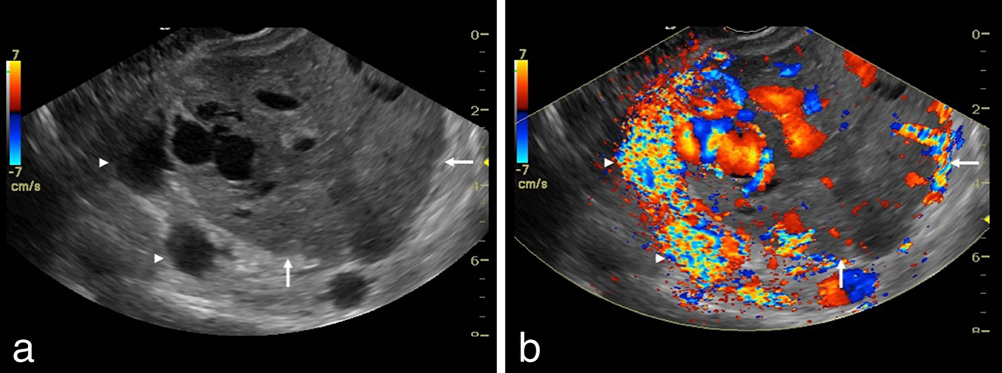
(a) Sagittal greyscale view of the pelvis shows a predominantly solid mass (arrows) with large internal vessels as well as a large vessel (arrowheads) draping over the superior margin mass. (b) Corresponding colour Doppler view demonstrates marked flow within the large vessels. The large vessel (arrowheads) draping over the superior margin of the mass (arrows) exhibits prominent aliasing, reflecting high velocity.

**Figure 2.**
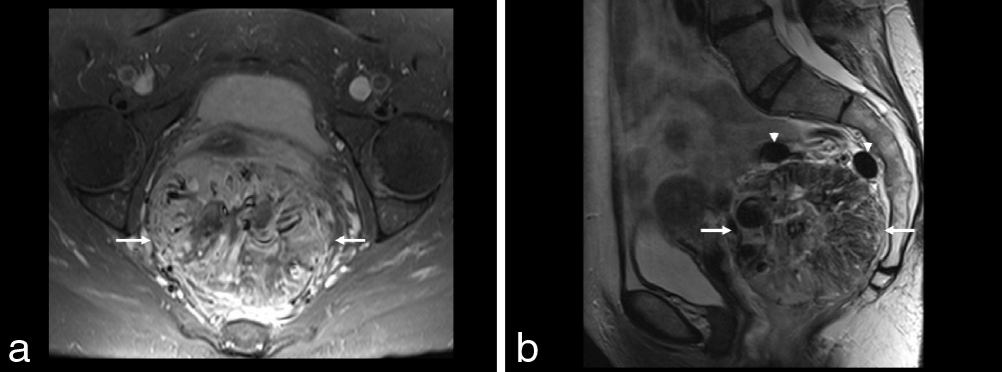
(a) Axial *T*_1_ fat saturation post-contrast demonstrates a large rounded vascular mass (arrows) with central areas of non-enhancement. (b) Sagittal *T*_2_ weighted MRI of the pelvis shows a large heterogeneous predominantly hypointense round mass (arrows) in the pelvis with numerous rounded areas which represent flow voids within prominent draining veins (arrowheads).

**Figure 3.**
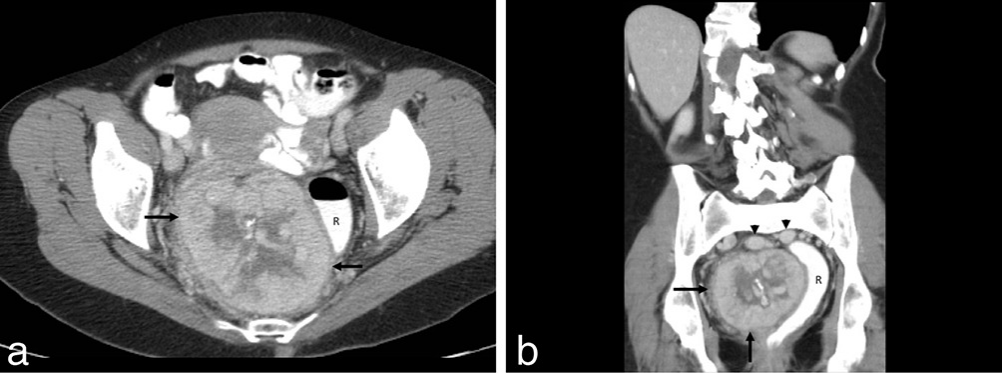
(a, b). Axial (a) and coronal (b) contrast-enhanced CT shows an 8 cm presacral soft tissue mass (arrows) with central calcifications and hypodensity, likely reflecting central necrosis. The mass causes significant deviation of the rectum (R) to the left, better appreciated on the coronal reformat (b). Prominent draining veins (arrowheads in b) are well seen abutting the cranial aspect of the mass.

The patient was referred for ultrasound, which showed a solid, vascular mass posterior to the uterus (Figure 1a,b). MRI redemonstrated the heterogeneous, hypervascular mass posterior to the uterus in the deeper pelvic rim. There was an intermediate signal on *T*_1_ weighted images and an internal hyperintensity consistent with haemorrhage. Numerous rounded areas representing flow voids within large vessels were also seen (Figure 2a,b). A subsequent CT scan showed a well-defined, round, soft tissue pelvic midline mass, measuring 8.1 × 8.5 cm, with punctate central calcifications and large draining veins (Figure 3a,b) that drained into the inferior mesenteric vein. The mass compressed the rectum and deviated it to the left without narrowing its lumen. Colonoscopy revealed mild external compression of the rectum due to the known pelvic mass. There was no evidence of lymphadenopathy or distant metastatic disease on any radiological study.

## Treatment

The patient underwent surgical resection of the pelvic tumour. Intraoperatively, the mass was found to adhere to the rectum and the posterior vagina near the cervix. Accordingly, she underwent en bloc resection of the cervix, partial vaginectomy, partial proctectomy, coloanal anastomosis and a loop-diverting ileostomy. There was an intraoperative blood loss of 1.5 l that was attributed to increased vascularity and large vessels throughout the tumour. All bleeding stopped as soon as the tumour was removed.

Gross examination revealed a well-encapsulated, tan-brown mass measuring 10.0 × 8.0 × 6.5 cm. There were areas of necrosis seen on the cut surfaces. The tumour involved the muscularis propria of the colon and perivaginal fibroconnective tissue, but there was no evidence of invasion into the rectal or vaginal mucosa. The resected margins were negative. Histological findings demonstrated a spindle cell proliferation with staghorn vasculature and short, stubby interweaving nuclei. No mitotic figures or atypical cells were seen. Immunohistochemical stains were positive for CD34 and STAT-6, but negative for S100, desmin, CKMIX and ERG.

On the basis of these pathological findings, benign pelvic solitary fibrous tumour (SFT) was diagnosed. The patient recovered without complications and was symptom free at 10 months follow-up, with no evidence of residual or recurrent disease on MRI. No adjuvant therapy was pursued. Since fibrous tumours can metastasize to the lung and liver, the patient will require ongoing imaging surveillance.

## Discussion

SFT is a rare spindle cell neoplasm that accounts for approximately 2% of all soft tissue tumours.^1^ It has most frequently been described in the thoracic cavity arising from the pleura. Extrapleural SFT occurs in a wide range of anatomic sites, including the salivary glands, thyroid, liver, adrenals, bladder and testes.^2^ The abdominopelvic cavity appears to be one of the more common locations for an extrapleural SFT.^3^

Regardless of anatomic location, the defining oncogenic driver mutation of SFTs appears to be a chromosomal rearrangement adjoining NAB2 with STAT6. The genetic fusion product combines the EGR-binding domain of NAB2 with the activation domain of STAT6.^4,5^ However, outside of this unifying molecular hallmark, SFTs possess highly variable cellular signalling components, mitotic indexes and extracellular matrix. Currently, they are classified into three main variants: usual, malignant and dedifferentiated SFT.^6^

Most SFTs are benign, slow-growing lesions with well-defined borders. In these cases, patients are usually asymptomatic or present with symptoms attributable to the compression of adjacent structures. Local recurrence or metastasis develops in 12–22% of cases.^7^ Given its malignant potential, the recommended first-line treatment for localized SFT is complete surgical resection. In patients with highly vascular SFT, pre-operative embolization may be performed as a precautionary measure to minimize intraoperative haemorrhage.^8^ Adjuvant chemoradiotherapy is not widely practiced or accepted as standard of care. Patients who ultimately develop locally recurrent or metastatic disease have a poor prognosis. Currently, there is limited data to support a chemoradiation strategy for this population, in particular because the low incidence of SFT precludes large randomized studies. In retrospective analyses, conventional chemotherapeutic agents have demonstrated minimal efficacy.^9–11^ However, antiangiogenic agents and other tyrosine kinase inhibitors have shown encouraging results in case series, suggesting a potential role for molecular targeted therapy.^12^

On CT, pelvic SFTs appear as well-circumscribed masses that often compress adjacent tissues and organs. Large pelvic SFTs have been reported to result in large bowel obstruction and various urinary symptoms including urinary retention and bilateral hydronephrosis.^13,14^ In our patient, the large tumour caused marked deviation of the rectum; however, it did not cause constipation or urinary symptoms. Scattered intratumoral foci of hypoenhancement or non-enhancement usually represent regions of necrosis, haemorrhage or cystic change. These findings are more common in relatively large masses; smaller lesions, by comparison, typically demonstrate homogeneous enhancement.^15^ The radiological distinction between benign and malignant SFTs is difficult to establish. However, this may be inferred from a CT scan by defining the local extent of disease and presence of distant metastases. In this patient, CT findings were rather characteristic; they showed a well-circumscribed, hypervascular mass that exerted pressure effect and displaced the rectum. Additional typical findings included central hypoenhancing and non-enhancing areas within the tumour, representing necrosis or cystic changes. Calcifications were also present and are a rare feature that can be seen in large benign or malignant tumours.

MRI is a useful complementary test for characterizing the primary lesion and assessing the extent of disease burden. On *T*_1_ weighted images, SFT usually appears as an intermediate, heterogeneous signal intensity. Areas of subacute haemorrhage can be identified by *T*_1_ weighted signal enhancement. On *T*_2_ weighted images, flow voids can be seen as areas of heterogeneous low-signal intensity, as was the case in our patient.^16^ Gadolinium-enhanced, fat-suppressed *T*_1_ weighted MR shows intense heterogeneous enhancement of the pelvic mass in the arterial phase, with progressive enhancement in the venous phase, which are findings consistent with the predominant fibrous content of the tumour.^15^

On ultrasound examination, pelvic SFT can often be seen as a hypoechoic mass, but occasionally it is heterogeneous. The latter finding corresponds to the heterogeneity identified using other imaging modalities and likely represents areas of myxoid degeneration. Since SFT is a highly vascular neoplasm, the lesion exhibits flow during Doppler imaging, as was seen in our patient.^17^

Additional complementary imaging studies include angiography and fludeoxyglucose positron emission tomography (PET) CT. On angiography, pelvic SFT typically appears as a very vascular mass with prominent blood vessels. Dilated arteries and early visualization of veins (arteriovenous shunt) may also be seen. PET-CT may show heterogeneous areas of increased uptake that correspond to hypercellular areas within the tumour.^15^

Although radiological findings may suggest a diagnosis of SFT, they are often non-specific. Histopathological and immunohistochemical features can aid in determining the correct diagnosis. SFT cells characteristically stain positive for CD34, CD99, Bcl-2 and vimentin, and negative for S100, actin and keratin.^18^ Importantly, many other spindle cell tumours stain negative for CD34. On gross examination, SFTs may contain areas of intratumoral haemorrhage, necrosis, cystic changes and calcification.^19^

There are several important pathological features that portend recurrence and metastasis. These include tumour size >10 cm, positive surgical margins and foci of haemorrhage and necrosis. Histological changes such as hypercellularity, increased mitotic activity (>4 mitotic figures per 10 high-power fields) and nuclear pleomorphism are also associated with increased malignant potential. Nevertheless, the presence or absence of these histopathological findings does not reliably predict clinical outcome. Recurrence and metastasis may develop in patients who were previously thought to have benign lesions. This warrants long-term, post-operative surveillance for all patients with SFTs.^20^

## Learning points

1. Pelvic SFT presents as a large (average 10 cm), well-circumscribed, hypervascular mass that often compresses adjacent tissues and organs.
2. On CT, the mass often appears as a well-defined, intensely enhancing mass in the pelvis with central hypoenhancing areas. Central calcifications are seen in larger masses.
3. On *T*_1_ weighted MR images, SFT usually shows an intermediate, heterogeneous signal intensity. On *T*_2_ weighted images, flow voids can be seen as areas of heterogeneous low signal intensity.

## Consent

Written informed consent was obtained from the patient for publication of this case report, including accompanying images.
